# Pivotal Role for Cxcr2 in Regulating Tumor-Associated Neutrophil in Breast Cancer

**DOI:** 10.3390/cancers13112584

**Published:** 2021-05-25

**Authors:** Colin Timaxian, Christoph F. A. Vogel, Charlotte Orcel, Diana Vetter, Camille Durochat, Clarisse Chinal, Phuong NGuyen, Marie-Laure Aknin, Françoise Mercier-Nomé, Martin Davy, Isabelle Raymond-Letron, Thi-Nhu-Ngoc Van, Sarah D. Diermeier, Anastasia Godefroy, Magali Gary-Bobo, Franck Molina, Karl Balabanian, Gwendal Lazennec

**Affiliations:** 1 CNRS, SYS2DIAG-ALCEDIAG, Cap Delta, 1682 rue de la Valsière, 34184 Montpellier, France; timax.co@gmail.com (C.T.); charlotte.orcel15@gmail.com (C.O.); diana.vetter@sys2diag.cnrs.fr (D.V.); camille.durochat@gmail.com (C.D.); chinal.clarisse@gmail.com (C.C.); phuongntt995@gmail.com (P.N.); martin.davy@sys2diag.cnrs.fr (M.D.); thi-nhu-ngoc.van@sys2diag.cnrs.fr (T.-N.-N.V.); franck.molina@sys2diag.cnrs.fr (F.M.); 2 CNRS, GDR 3697 Microenvironment of Tumor Niches, Micronit, France; karl.balabanian@inserm.fr; 3 Center for Health and the Environment, University of California, 1 Shields Avenue, Davis, CA 95616, USA; cfvogel@ucdavis.edu; 4 CNRS, Institut Paris Saclay d’Innovation Thérapeutique, Université Paris-Saclay, Inserm, 92296 Châtenay-Malabry, France; marie-laure.aknin@u-psud.fr (M.-L.A.); francoise.mercier-nome@universite-paris-saclay.fr (F.M.-N.); 5 Department of Histopathology, National Veterinary School of Toulouse, 31076 Toulouse, France; i.raymond@envt.fr; 6 Platform of Experimental and Compared Histopathology, STROMALab, UMR UPS/CNRS 5223, EFS, Inserm U1031, 31076 Toulouse, France; 7 Department of Biochemistry, University of Otago, Dunedin 9016, New Zealand; sarah.diermeier@otago.ac.nz; 8 IBMM, University of Montpellier, CNRS, ENSCM, 34093 Montpellier, France; anastasia.gdy@gmail.com (A.G.); magali.gary-bobo@inserm.fr (M.G.-B.); 9 Institut de Recherche Saint-Louis, Université de Paris, EMiLy, Inserm U1160, 75010 Paris, France

**Keywords:** chemokine receptors, breast cancer, Cxcr2, neutrophils, tumor microenvironment

## Abstract

**Simple Summary:**

Chemokines present in the tumor microenvironment are essential for the control of tumor progression. We show here that the knock-down of Cxcr2 in PyMT animals led to an increased growth of the primary tumor and lung metastasis. The analysis of tumor content of PyMT-Cxcr2−/− animals highlighted an increased infiltration of tumor associated neutrophils (TANs), mirrored by a decreased recruitment of tumor associated macrophages (TAMs) compared to PyMT animals. Analysis of PyMT-Cxcr2−/− TANs revealed that they lost their killing ability compared to PyMT-Cxcr2+/+ TANs and that they had a more pronounced pro-tumor TAN2 profile compared to PyMT TANs. PyMT-Cxcr2−/− TANs displayed an up-regulation of the pathways involved in reactive oxygen species (ROS) production and angiogenesis and factors favoring metastasis, but reduced apoptosis. In summary, our data reveal that a lack of Cxcr2 provides TANs with pro-tumor effects.

**Abstract:**

Chemokines present in the tumor microenvironment are essential for the control of tumor progression. We show here that several ligands of the chemokine receptor Cxcr2 were up-regulated in the PyMT (polyoma middle T oncogene) model of breast cancer. Interestingly, the knock-down of Cxcr2 in PyMT animals led to an increased growth of the primary tumor and lung metastasis. The analysis of tumor content of PyMT-Cxcr2−/− animals highlighted an increased infiltration of tumor associated neutrophils (TANs), mirrored by a decreased recruitment of tumor associated macrophages (TAMs) compared to PyMT animals. Analysis of PyMT-Cxcr2−/− TANs revealed that they lost their killing ability compared to PyMT-Cxcr2+/+ TANs. The transcriptomic analysis of PyMT-Cxcr2−/− TANs showed that they had a more pronounced pro-tumor TAN2 profile compared to PyMT TANs. In particular, PyMT-Cxcr2−/− TANs displayed an up-regulation of the pathways involved in reactive oxygen species (ROS) production and angiogenesis and factors favoring metastasis, but reduced apoptosis. In summary, our data reveal that a lack of Cxcr2 provides TANs with pro-tumor effects.

## 1. Introduction

Tumor cell interactions with the tumor microenvironment play a crucial role in tumor initiation, progression, metastasis, and response to therapies. Tumor microenvironment comprises not only immune cells, such as B and T lymphocytes, dendritic cells, NK cells, macrophages and neutrophils, but also mesenchymal stromal/stem cells (MSCs), cancer associated fibroblasts (CAFs) and endothelial cells, making the interactions between them relatively complex [1,2,3]. Tumor, immune, and stroma cells communicate with each other either by direct contacts or by the secretion of vesicles or soluble factors such as growth factors, cytokines, or chemokines [4].

Chemokines and their cognate G-protein coupled receptors are actively controlling immune responses primarily to recruit leukocytes to sites of inflammation, but are also modulating homeostatic functions [5]. If chronic inflammation found in cancer has for a long time been considered as an attempt of the host to eliminate the cancer, it is now believed that inflammation can also be crucial in tumor progression and involves in particular what can be called a “chemokine storm” [6,7]. These chemokines can be secreted by all types of cells, including cancer cells themselves, but the nature and the number of chemokines produced varies with the type of cells, their environment, and the stimuli that they receive from other cells.

We and others have shown that Cxcr2 ligands (Cxcl1, 2, 3, 5, 6, 7, 8) contribute to the aggressiveness of several types of cancers including breast [7,8,9,10,11,12]. CXCR2 ligands can be directly secreted by breast cancer cells, but can also be produced by endothelial cells, MSCs, or CAFs [3,8,11,12].

We have recently shown that CXCR2 was expressed by neutrophils in breast cancer samples and that CXCR2 was associated with a lower risk of relapse in patients [13]. Moreover, high CXCR2 levels associated with triple-negative breast patients with a better prognosis [14]. At steady state, CXCR2 has also been shown to be expressed mainly by neutrophils and to a lesser extent by endothelial cells [15] and is an essential regulator of neutrophil action. Previous work on CXCR2 function has shown that it was a pro-angiogenic receptor [16], but it has now been demonstrated, using Cxcr2 knock out (KO) animals, that CXCR2 is also involved in chronic obstructive pulmonary disease [17], wound healing [18], resistance to infections [19], myelin repair [20], metabolism [21] or reproduction under microbiota influence [22]. KO animals for Cxcr2 exhibit a lymphadenopathy due to an increased number of B lymphocytes and a splenomegaly owing to an accumulation of metamyelocytes and neutrophils. In addition, impairment in the recruitment of neutrophils has also been observed during acute inflammatory conditions [23].

It becomes increasingly clear that neutrophils and macrophages play a major role in tumor progression. In particular, tumor associated neutrophils (TANs) and tumor associated macrophages (TAMs) have begun to be characterized in different types of cancers. The variety of effects and plasticity of such cells has led to the definition of anti-tumor cells (TAN1 and TAM1) and pro-tumor cells (TAN2 and TAM2) [24,25,26,27,28].

Neutrophils, which are produced in the bone marrow, are the most abundant population of leukocytes in the circulation and can be rapidly mobilized to infection sites by extravasation from the circulation to the target tissues [29]. They are involved in host-defense, by engulfing and killing invading microorganisms. such as bacteria and fungi. Eradicating infections involves different mechanisms including phagocytosis, release of reactive oxygen species (ROS) and granular proteins and the production of cytokines. In addition, the release of extracellular traps (NETs) contributes to the clearing [30]. The classification of the different types of neutrophils present in human or mouse models harboring a tumor remains controversial [28]. First, the phenotype of the neutrophils found in different locations, including the tumor itself, peripheral organs involved in the generation or maturation of neutrophils (bone marrow, spleen), the circulation, or sites of metastasis such as the lung will be clearly different. Second, when focusing on the tumor itself, in addition to anti-tumor TAN1 and pro-tumor TAN2, one must also consider myeloid-derived suppressor cells (G-MDSCs). These immature myeloid cells display immunosuppressive properties and are now divided in granulocytic MDSCs (G-MDSCs) and monocytic MDSCs (M-MDSCs) [25,26,28].

As neutrophils are also increasingly recognized as key modulators of tumor progression and highly express the chemokine receptor Cxcr2, we decided to evaluate the effect of knocking-down its expression in the murine breast cancer model PyMT [31]. In the present study, loss of expression of Cxcr2 leads to a pro-tumorigenic effect with not only an increase in the growth of the primary tumor, but also a higher rate of development of lung metastases. Characterization of intra-tumor content of PyMT-Cxcr2−/− mice showed a higher number of infiltrating TANs, but a reduced number of TAMs. Moreover, Cxcr2−/− TANs exhibited a lower ability to kill tumor cells. By performing a transcriptomic analysis, we showed that Cxcr2−/− TANs had a more pronounced TAN2 phenotype than WT TANs and that multiple pathways of neutrophil action were dysregulated, suggesting that Cxcr2 could be involved in TAN plasticity.

## 2. Materials and Methods

### 2.1. Animal Models and Housing

All animal experiments conformed to our animal protocols that were reviewed and approved by the Institutional Animal Care and Use Committee. Cxcr2−/− mice [23] and PyMT [31] were obtained from the Jackson Laboratory. PyMT-CXCR2−/− mice were backcrossed in FVB genetic background for more than 12 generations. PyMT and control (WT) mice were also in a FVB background. All mice were genotyped to confirm the presence or not of PyMT transgene and CXCR2 allele. All mice were housed in a SOPF (specific and opportunistic pathogen free) animal facility.

### 2.2. Isolation of Cells

Cells from the bone marrow were isolated by centrifugation from the femurs and tibias of the animals, whereas spleens were mashed on 100 µm nylon cell strainer. After centrifugation, red blood cells were eliminated by treatment with ACK buffer (0.155 mM NH4Cl, 1 mM KHCO3, 0.1 mM EDTA) and filtered on a 40 µm nylon cell strainer. Cells from tumors of mammary glands 4 and 9 were isolated following a modified protocol from Dr J. Stingl [32]. Briefly, mammary glands or mammary tumors were minced with scalpels and digested using Tumor Dissociation Kit (Miltenyi, Paris, France) following manufacturer recommendations. After ACK treatment, cells were filtered on a 40-µm nylon cell strainer.

For neutrophil isolation, a first enrichment with EasySep™ Mouse CD11b Positive Selection kit (StemCell technologies, Grenoble, France) was performed followed by cell sorting of CD45+ CD11b+ Ly6G+ F4/80− cells (for neutrophils) and CD45+ Ly6G− F4/80+ (for macrophages) on an ARIA IIu FACS sorter (Becton Dickinson, Le Pont-de-Claix, France).

### 2.3. Flow Cytometry

Flow cytometry experiments were performed with the following conjugated antibodies from Biolegend (Ozyme, Saint-Cyr-l’École, France): anti-mouse CD11b (clone M1/70), CD11c (clone N418), CD45 (clone 30−F11), Cxcr2 (clone SA044G4), F4/80 (clone BM8), Ly6G (clone 1A8), Ly6C (clone HK1.4). Flow analysis was performed on live singlets with a LSR II Fortessa flow cytometer (Becton Dickinson, Le Pont-de-Claix, France). Data were analyzed using FlowJo (Tree Star, Ashland, OR 97520, USA).

### 2.4. Lung Metastasis Evaluation

Lung metastasis analyses were performed using 3−μm−thin sections from formalin−fixed paraffin−embedded tissue blocks. Counterstaining was performed using Flex Hematoxylin (Dako−Agilent, Les Ulis, France) followed by washing the slides under tap water for 5 min. Finally, slides were mounted with a coverslip after dehydration. The NanoZoomer slide scanner system (Hamamatsu Photonics, Massy, France) was used to digitalize glass slides at the ×40 objective. The number of metastases was counted and adjusted to the total surface of each lung slide.

### 2.5. PyMT Killing

To evaluate tumor cell killing by neutrophils, we used PyMT breast cancer cells stably transfected with a CMV−luciferase reporter (PyMT−luc) [33]. 10,000 PyMT−luc cells were cultured alone or in the presence of 10^5^ neutrophils isolated either from the spleen, the bone marrow or tumors. After 24 h, non−adherent cells were washed away with PBS, the number of surviving cells was evaluated using a luciferase reporter assay system (Promega, Madison, WI, USA), and the percentage of killing was calculated.

### 2.6. RNA Extraction and Reverse Transcriptase, Quantitative PCR

Total RNA was isolated using TRIzol reagent (ThermoFisher, Illkirch, France), as described by the manufacturer. Reverse transcription was performed with 1µg of total RNA using random primers and with M−MLV enzyme (ThermoFisher, Illkirch, France). Real time quantitative PCR was performed with SYBR green Master Mix (Roche, Meylan, France), on a Light Cycler 480 instrument (Roche) as previously described [22]. Ribosomal protein S9 (rS9) and GAPDH were used as an internal control. The sequence of the primers used in this study is indicated in Table S1. Results are expressed as N−fold differences in target gene expression relative to the internal control gene and termed “mRNA expression”, determined as mRNA expression = 2 − Δ Ctsample, where the Δ Ct value of the sample was determined by subtracting the Ct value of the target gene from the Ct value of the average of the internal control genes. Target genes were considered to be non−detectable when the Ct value was above 35.

### 2.7. RNA−Seq Data Processing

RNA integrity and quality were verified using RNA ScreenTape kit and Tapestation 2200 apparatus from AGILENT (Les Ulis, France). cDNA libraries were synthesized using NEBNext^®^ rRNA Depletion and Ultra™ II Directional RNA Library Prep Kit (New England Biolabs, Evry-Courcouronnes, France). Library quality was checked on Tapestation 2200 apparatus from AGILENT (Les Ulis, France) with DNA 1000 ScreenTape. Samples were sequenced on Novaseq 6000 (Illumina, 91030 Evry, France) with an average sequencing depth of 30 million of paired−end reads. Length of the reads was 150 bp. Each 24 Plex Samples was sequenced on one Illumina SP FlowCell (2 × 800 million of 150 bases reads). Raw sequencing data were quality−controlled with the FastQC program. Low−quality reads were trimmed or removed using Trimmer (minimum length: 120 bp). Reads were aligned to the mouse reference genome (mm10 build) with the Star tool. Gene counts were obtained by read counting software Htseq. Normalization and differential analysis were performed with the DESeq2 package with Benjamini–Hochberg FDR multiple testing correction (*p* < 0.05; 1.5−fold or higher change) comparing WT and KO animals. The data discussed in this publication have been deposited in NCBI’s Gene Expression Omnibus [34] and are accessible through GEO Series accession number GSE164766 (https://www.ncbi.nlm.nih.gov/geo/query/acc.cgi?acc=GSE164766, accessed on 21 May 2021).

### 2.8. Bioinformatic Analysis

To assess biological interpretation of the most differentially expressed genes, we used Gene ontology (GO) enrichment analysis. A gene set enrichment analysis (GSEA) was performed using signatures from GSEA collections for biological process or molecular function. In addition, gene sets were constructed using data from Shaul et al. [35], Zilionis et al. [36]. A normalized enrichment score (NES) was calculated for each gene set and only gene sets with an adjusted *p* value < 0.05 were selected.

### 2.9. Statistics

Statistical analyses were carried out using unpaired Mann–Whitney test.

## 3. Results

### 3.1. Cxcr2 Ligands Levels Increase in Mouse Breast Cancers

**Figure 1:**
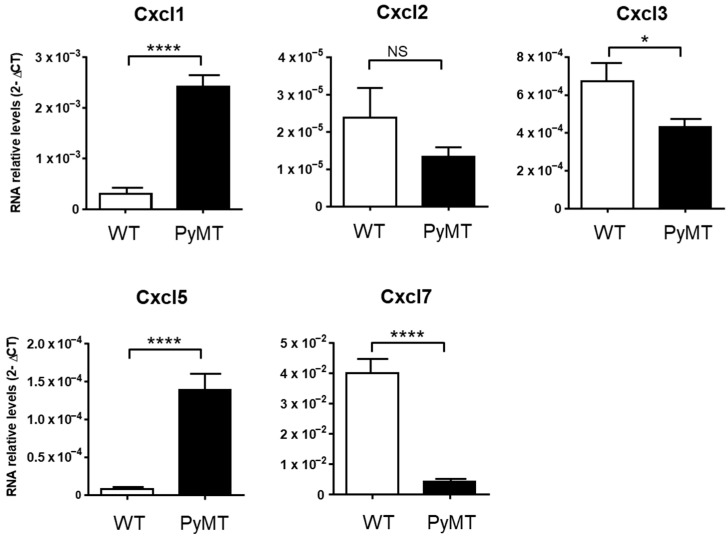
Cxcl1 and Cxcl5 levels increase in PyMT tumors. Measure of RNA levels by real−time PCR of Cxcl1, 2, 3, 5 and 7 in the mammary gland of WT or the tumor of PyMT animals of 10 weeks. Results represent the mean ± SEM of at least 14 animals (Mann−Whitney test, NS: non−significant, * *p* < 0.05, **** *p* < 0.0001).

We previously showed that Cxcr2 ligands were present at higher levels in more aggressive forms of human breast cancer [8,10,12]. We decided to evaluate if this was also the case in the murine model of breast cancer PyMT [31]. The quantification of Cxcr2 ligands showed that Cxcl1 and Cxcl5 RNA levels increased in the tumor of PyMT animals compared to wild−type (WT) mammary gland (Figure 1). This was not the case for all Cxcr2 ligands as Cxcl2 RNA levels were not significantly affected, whereas Cxcl3 and Cxcl7 decreased in PyMT tumors compared to the mammary gland of WT animals.

In another model of murine breast cancer, MMTV−Neu (Mouse Mammary Tumor Virus—Neu oncogene) [37], we observed an increase of Cxcl1, Cxcl2, Cxcl3, and Cxcl5 levels in the tumor of MMTV−Neu animals compared to the mammary gland of WT animals (Figure S1). This confirmed the involvement of Cxcr2 ligands and notably Cxcl1 and Cxcl5 in breast carcinogenesis, and led us to investigate the effects of invalidating Cxcr2.

### 3.2. Cxcr2 Is Expressed by Neutrophils

We first determined which types of cells were expressing high levels of CXCR2 in PyMT tumors. We compared Cxcr2 staining by FACS in PyMT and PyMT−Cxcr2−/− tumors (Figure S2). This shows that epithelial cancer cells (CD45− Epcam+ cells) did not express Cxcr2, whereas in the CD45 immune population, Cxcr2 was expressed only in CD11b+ granulocytic cells. When looking in more detail at which type of CD11b+ cells were expressing Cxcr2, we observed that neutrophils (CD45+ CD11b+ Ly6G+ cells) but not macrophages (CD45+ CD11b+ Ly6G− F4/80+ cells) were expressing high levels of Cxcr2, confirming that neutrophils represent the cells expressing the highest levels of Cxcr2.

### 3.3. Cxcr2 Knock−Down Accelerated Tumor Growth

**Figure 2:**
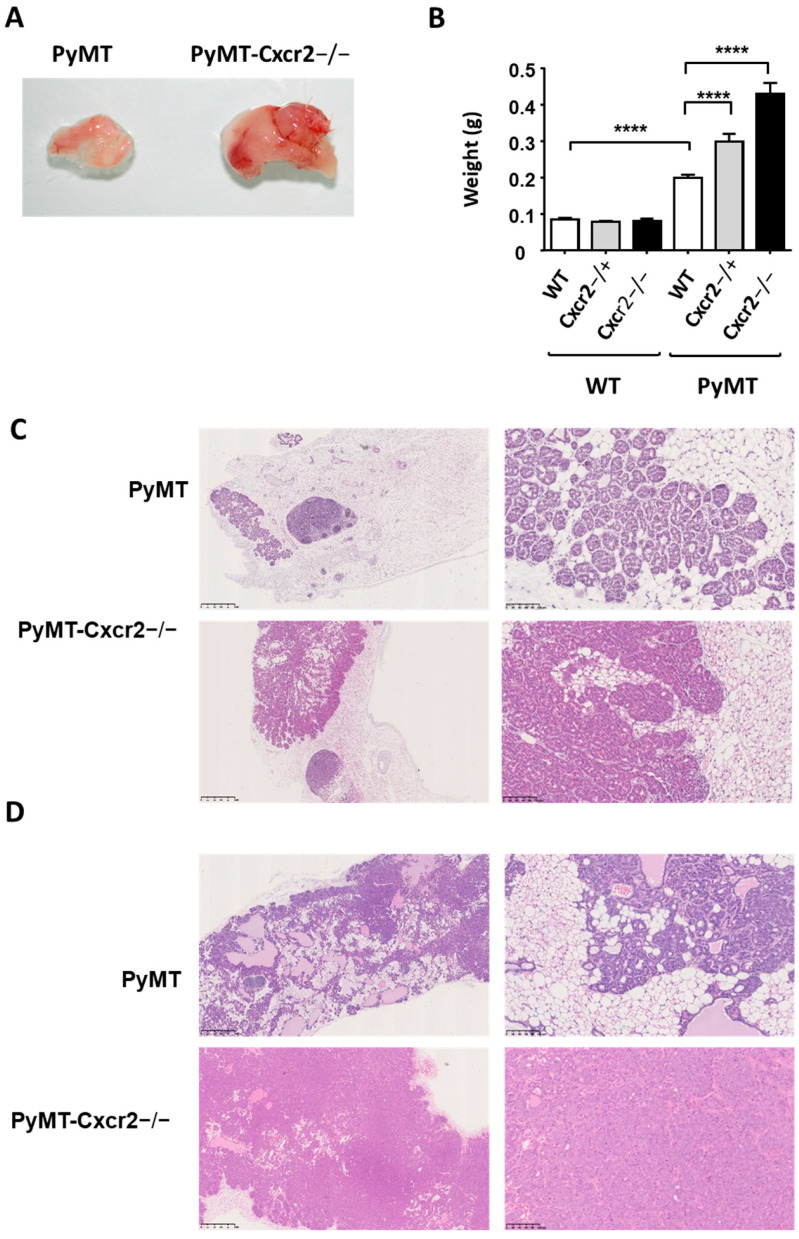
Knock out of Cxcr2 favors tumor growth of PyMT animals. (**A**). Representative images of PyMT and PyMT−Cxcr2−/− tumors of 10 weeks old animals. (**B**) Weight of 10 week old mammary gland of WT, Cxcr2−/+, Cxcr2−/− PyMT, PyMT−Cxcr2−/+ and PyMT−Cxcr2−/− mice. Results represent the mean ± SEM of at least 20 animals (Mann−Whitney test, **** *p* < 0.0001). (**C**) Histology of the mammary glands of PyMT and PyMT−Cxcr2−/−animals at 6 weeks. Representative images of hematoxylin−eosin stained mammary glands at a 2.5× magnification (left panel, scale bars: 1 mm) and 20× magnification (right panel, scale bars: 100 µm) are shown here. (**D**) Histology of the mammary tumors of PyMT and PyMT−Cxcr2−/− animals at 10 weeks. Representative images of hematoxylin−eosin stained mammary glands at a 2.5× magnification (left panel, scale bars: 1 mm) and 20× magnification (right panel, scale bars: 100 µm) are shown here.

We crossed Cxcr2 KO mice and PyMT mice (both in a FVB background) and first analyzed the rate of tumor growth in these animals. We observed that ten−week old PyMT−Cxcr2−/− mice developed bigger tumors than WT (about twice heavier than PyMT tumors) (Figure 2A,B). Interestingly heterozygous animals (PyMT−Cxcr2−/+) also exhibit tumor sizes in between the ones of PYMT WT and PyMT−Cxcr2−/− mice, showing that Cxcr2 expression level follows a gene–dose effect (Figure 2B).

Moreover, the increased growth of PyMT−Cxcr2−/− tumors could be seen early, as six−week−old animals showed a clear increase in mammary gland weight compared to PyMT animals (Figure S3). The difference in tumor size between PyMT−Cxcr2−/− and PyMT animals was persistent after twelve weeks (Figure S3). When looking at the histology of the tumors, at only six weeks, PyMT−Cxcr2−/− tumors showed higher penetrance of the mammary gland, with a large part of the gland affected compared to PyMT tumors (Figure 2C). At ten weeks of age, the entire gland was completely colonized by tumor cells in PyMT−Cxcr2−/− animals, whereas part of the mammary gland remained tumor−free for PyMT animals (Figure 2D).

We also sought to determine if the RNA levels of Cxcr2 ligands were affected in PyMT−Cxcr2−/− compared to PyMT tumors. We observed an up−regulation of Cxcl1 and Cxcl5, suggesting a mechanism of compensation for Cxcr2 loss (Figure S4). Overall, our data show that Cxcr2 ablation accelerates primary tumor growth in the mammary gland.

### 3.4. Increased Splenomegaly and Lung Metastasis in PyMT−Cxcr2−/− Compared to PyMT Animals

**Figure 3:**
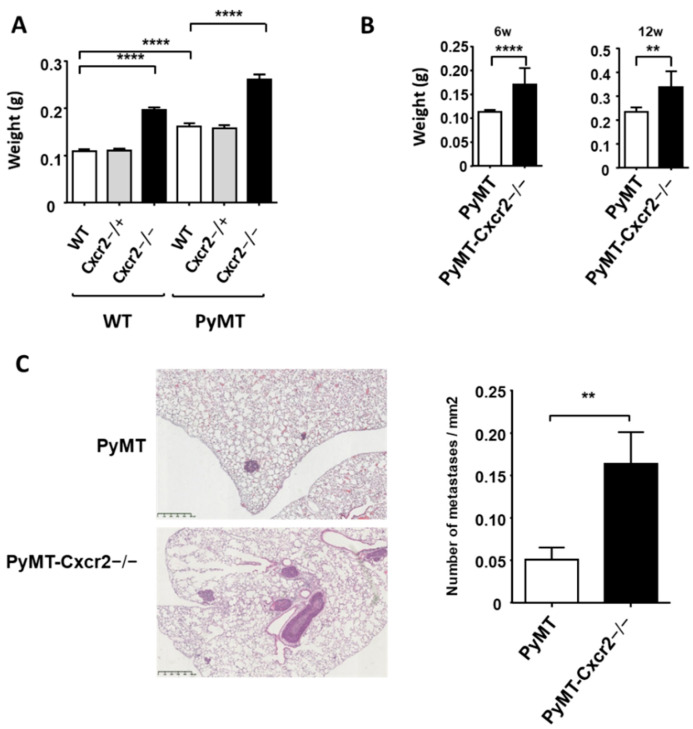
Knock out of Cxcr2 increases the size of the spleen and lung metastasis of PyMT animals. (**A**) Weight of 10 week old spleens of WT, Cxcr2−/+, Cxcr2 KO, PyMT, PyMT−Cxcr2−/+ and PyMT−Cxcr2−/− mice. Results represent the mean ± SEM of at least 14 animals (Mann−Whitney test, ** *p* < 0.01, **** *p* < 0.0001). (**B**) Weight of the spleens of 6 weeks (left panel) and 12 weeks (right panel) old PyMT and PyMT−Cxcr2−/− mice. Results represent the mean the mean ± SEM of at least 14 animals. (**C**) Left panel: Representative images of lung metastases in 12 weeks old PyMT and PyMT−Cxcr2−/− mice at a 5× magnification (right panel, scale bars: 500 µm). Right panel: Number of lung metastasis/mm^2^ observed in PyMT and PyMT−Cxcr2−/− mice of 12 weeks. Results represent the mean ± SEM of 15 animals.

The measure of the spleen of ten week−old mice showed that Cxcr2−/− animals had a clear increase in spleen size (Figure 3A), which is in agreement with the increased number of metamyelocytes and neutrophils in the spleen initially reported for these animals [23]. Moreover, the spleen in PyMT mice was less enlarged compared to a 50% increase in spleen weight of PyMT−Cxcr2−/− mice (Figure 3A). The difference in spleen size and weight between PyMT−Cxcr2−/− and PyMT animals was already visible at six weeks of age and was also maintained at twelve weeks (Figure 3B).

We next investigated lung metastasis in twelve−week−old animals and observed the appearance of a higher number of metastases in PyMT−Cxcr2−/− compared to PyMT WT animals (Figure 3C), demonstrating that Cxcr2 knock−down affects not only primary tumor growth and spleen size, but also distant metastasis.

### 3.5. PyMT−Cxcr2−/− Tumors Exhibit a Higher Content of Neutrophils, but Fewer Macrophages

**Figure 4:**
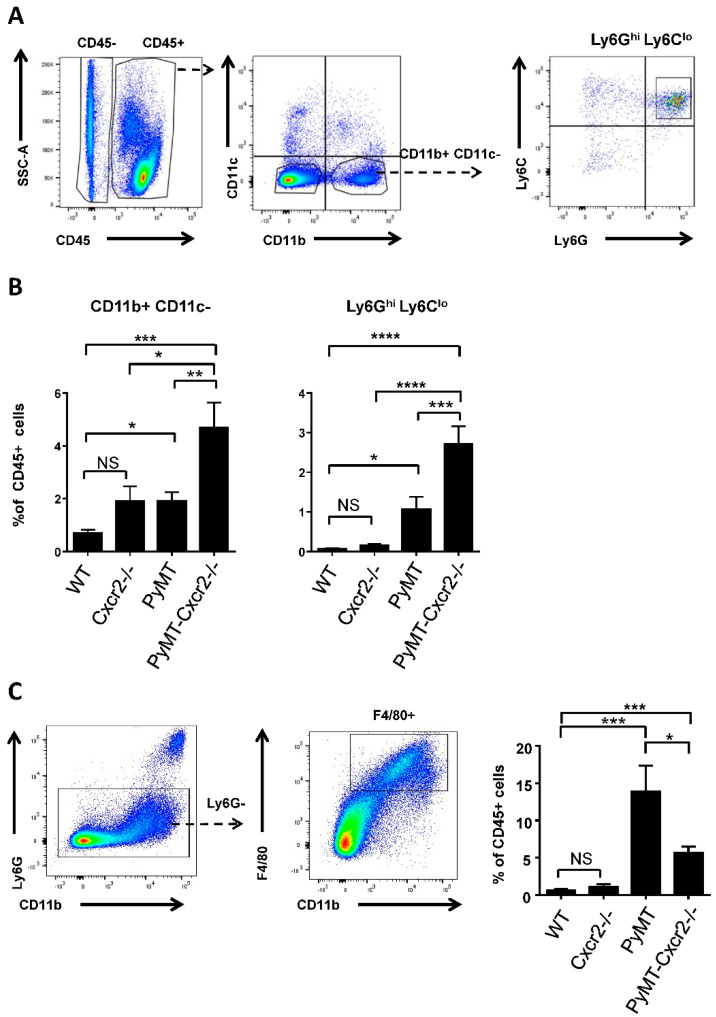
Cxcr2 deletion increases the recruitment of neutrophils but reduces macrophage infiltration in the tumors of PyMT animals. (**A**) Representative dot plots of the gating strategy of CD45+ CD11b+ CD11c−, CD45+ CD11b+ CD11c−Ly6G^hi^ Ly6C^lo^ cells in the mammary gland. (**B**) Quantification of the percentage of CD11b+ CD11c− cells (left panel) and of CD11b+ CD11c−Ly6G^hi^ Ly6C^lo^ (middle panel) (right panel) in the CD45+ fraction. Data represent the mean ± SEM of at least 7 animals (Mann−Whitney test, NS: non−significant, * *p* < 0.05, ** *p* < 0.01, *** *p* < 0.001, **** *p* < 0.0001). (**C**) Left panel: Gating strategy of CD45+ CD11b+ Ly6G− F4/80+ macrophages. Right panel: Bar graphs showing the percentage of CD11b+ Ly6G− F4/80+ macrophages in CD45+. Data represent the mean ± SEM of at least 7 animals (Mann−Whitney test, NS: non−significant, * *p* < 0.05, ** *p* < 0.01, *** *p* < 0.001, **** *p* < 0.0001).

To understand the reason why PyMT−Cxcr2−/− tumors grew more rapidly than PyMT tumors, we analyzed the tumor content in immune cells in both types of mouse strains. Due to the critical role of Cxcr2 in neutrophil function, we first looked at neutrophils in primary tumors by flow cytometry. We observed an increase in the percentage of myeloid cells (CD45+ CD11b+ CD11c−) in the mammary gland of PyMT mice compared to WT glands (Figure 4A,B, left panel, Figure S5A,B). Moreover, the percentage of CD11b+ CD11c− cells was further multiplied by more than two times in the tumors of PyMT−Cxcr2−/− mice. To be more specific in the evaluation of neutrophils, an additional gating on Ly6Ghi and Ly6Clo was performed (Figure 4A,B, left panel and Figure S5C), as defined earlier [38]. Nearly all CD11b+ CD11c− cells were Ly6Ghi Ly6Clo, and correspond to tumor associated neutrophils (Figure 4A and Table S2). There was a clear increase of CD11b+ CD11c− Ly6G^hi^ Ly6C^lo^ cells in the mammary gland of PyMT mice compared to WT glands and this percentage increased by nearly three−fold in the tumors of PyMT−Cxcr2−/− mice (Figure 4B, right panel). This demonstrates that one of the major differences between PyMT and PyMT−Cxcr2−/− tumors is the content of neutrophils in the tumor.

We next analyzed macrophage content in tumors, by gating the cells on CD11b+ Ly6G− F4/80+ (Figure 4C). We observed a strong increase of macrophages in PyMT tumors compared to WT glands. However, this was less pronounced in PyMT−Cxcr2−/− tumors (Figure 4C right panel).

We also evaluated the variations of neutrophil content in the blood, spleen and bone marrow. There was a strong increase in CD11b+ CD11c− Ly6G^hi^ Ly6C^lo^ cells in in the spleen of PyMT−Cxcr2−/− animals compared to PyMT animals (Figure S6A), which was similar to Cxcr2−/− animals suggesting that this increase was related to Cxcr2 inactivation and not to the presence of a tumor. No difference of CD11b+ CD11c− Ly6G^hi^ Ly6C^lo^ cells was found in blood between PyMT−Cxcr2−/− and PyMT animals (Figure S6A). Moreover, we also observed a modest increase of 1.5 fold of neutrophils in the BM of Cxcr2−/− and PyMT−Cxcr2−/− animals compared to WT animals (Figure S6A).

Concerning macrophages, we observed also a decreased number of CD11b+ Ly6G− F4/80+ in the blood, whereas the number of macrophages was not altered in the spleen of PyMT−Cxcr2−/− compared to PyMT animals (Figure S6B).

### 3.6. Tans Have a Distinct Transcriptome Profile Compared to BM (Bone Marrow) Neutrophils

We first sought to evaluate whether PyMT and PyMT−Cxcr2−/− TANs were different from immature neutrophils, by comparing their transcriptome to the one of WT BM neutrophils (Figure S6). The two types of TANs were clearly distinct from WT BM neutrophils and had about the same number of differentially regulated genes compared to WT BM neutrophils (Figure S7A–C). This highlights that the TANs of PyMT−Cxcr2−/− and PyMT mice are tumor−specific. The nature of these differentially expressed genes was yet not identical as 3023 genes were specifically differentially regulated in PyMT−Cxcr2−/− TANs and 1916 in PyMT TANs, as shown by Venn diagram analysis (Figure S7D).

### 3.7. Pymt−Cxcr2−/− Tans Exhibit a More Pronounced TAN2 Profile Compared to Pymt Tans

**Figure 5:**
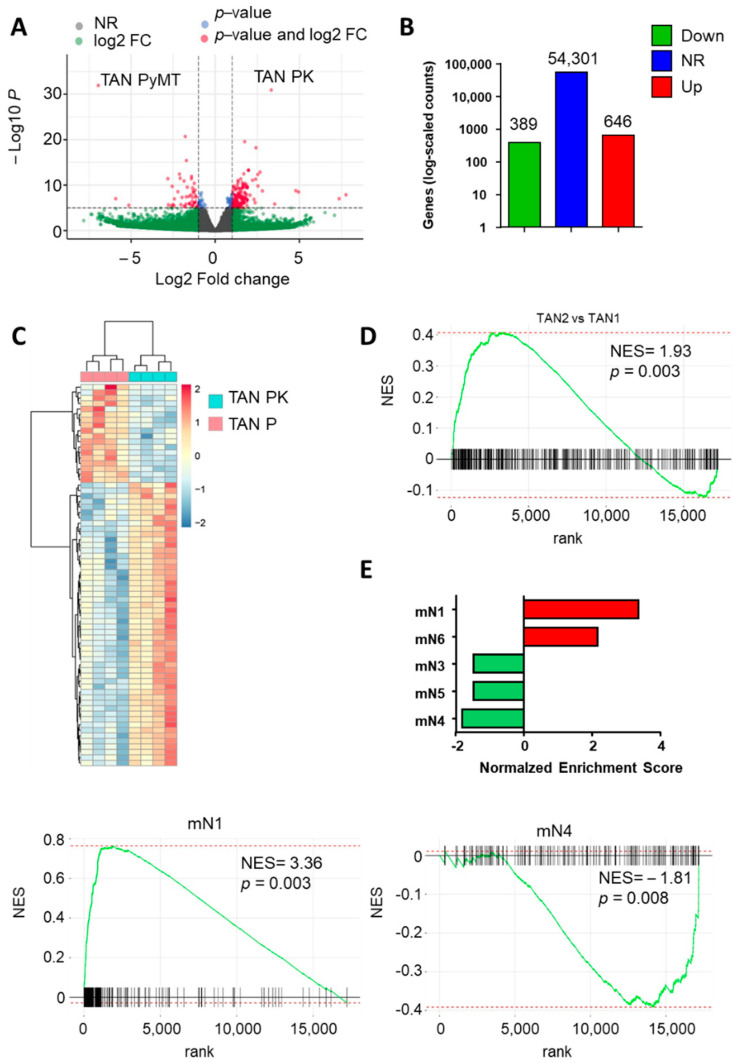
PyMT−Cxcr2−/− TANs have a more pronounced TAN2 profile. (**A**) Volcano plot showing the global changes in RNA expression patterns for Tumor neutrophils isolated from PyMT−Cxcr2−/− (TAN PK) versus PyMT (TAN PyMT) animals. Data represent analysis of cpm estimates with a log of fold change of more than 1.5 fold and *p* < 0.05 of 4 animals per group. Grey dots: NR: non−regulated genes; Green dots: genes with a log of fold change of more than 1.5 fold; blue dots: genes with a *p*−value < 0.05; red dots: genes with a log of fold change of more than 1.5 fold and *p* < 0.05. (**B**) Number of differentially regulated genes for the same analysis. Up: genes up−regulated in Tumor neutrophils isolated from PyMT−Cxcr2−/− versus PyMT animals. Down: down−regulated genes. NR: non regulated genes. (**C**) Heatmap of the comparison of Tumor neutrophils isolated from PyMT−Cxcr2−/− (TAN PK) versus PyMT (TAN PyMT) animals. (**D**) GSEA dataset of TAN2 over TAN1 enrichment (according to Shaul et al. [35]) found in neutrophils of PyMT−Cxcr2−/− versus PyMT animals. FDR < 0.05. (**E**) Normalized enrichment score (NES) after GSEA analysis of the transcriptome of tumor neutrophils isolated from PyMT−Cxcr2−/− versus PyMT animals according to Neutrophil classification of Zilionis et al. [36]. Bottom: GSEA dataset of mN1 and mN4 neutrophil enrichment. FDR < 0.05.

To identify the differences between the two types of TANs, we next directly compared PyMT−Cxcr2−/− and PyMT TANs transcriptomes (Figure 5). The two types of TANs display a number of different features as shown by volcano plot, numbers of differentially regulated genes and heatmap (Figure 5A–C respectively). We next compared our gene expression signatures to the one of Shaul et al. [35] to define TAN1 and TAN2. Interestingly, PyMT−Cxcr2−/− TANs showed a more pronounced TAN2 profile compared to PyMT TANs as shown by GSEA (Gene Set Enrichment Analysis) analysis (Figure 5D and Table S3). To have a better idea of the nature of the TANs found in the two types of animals, we compared their transcriptomic signature with the one defined by Zilionis et al. using single cell RNAseq analysis of neutrophils found in a murine model of lung cancer [36]. PyMT−Cxcr2−/− TANs were enriched in mN1 and mN6 neutrophils but showed reduced levels of mN3, mN4 and mN5 neutrophils, suggesting that they contain two types of neutrophils: some with the most advanced phenotypes towards tumor specific TANs (mN6) but also more immature neutrophils (mN1) that could reflect less tumor−specific neutrophils (Figure 5E and Table S4).

### 3.8. PyMT−Cxcr2−/− TANs Show Alterations in Key Pathways

**Figure 6:**
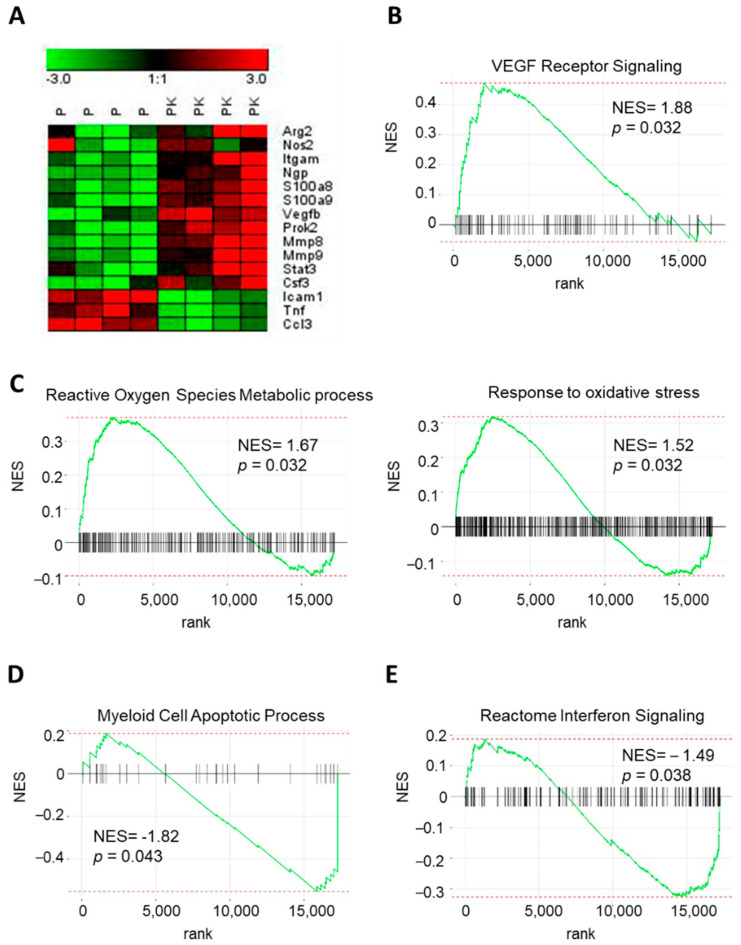
PyMT−Cxcr2−/− TANs display a particular biology. (**A**) Heatmap of key regulated genes between TAN PyMT (*p*) and TAN PyMT−Cxcr2−/− (PK) performed with Genesis software [40]. (**B**) Normalized enrichment score (NES) after GSEA analysis of the transcriptome of tumor neutrophils isolated from PyMT−Cxcr2−/− versus PyMT animals. FDR < 0.05. Increased enrichment of VEGF Receptor Signaling according to GSEA analysis of BP in PyMT−Cxcr2−/− versus PyMT TANs. (**C**) Enrichment in Reactive Oxygen Species Metabolic Process (left panel) and Response to oxidative stress (right panel) GO pathways in PyMT−Cxcr2−/− versus PyMT TANs. (**D**) Inhibition of Myeloid Cell Apoptotic Process according to GSEA analysis of BP. (**E**) Inhibition of Interferon signaling Reactome in PyMT−Cxcr2−/− versus PyMT TANs.

We focused our attention on key genes known to be important to define TAN2 and TAN1 properties [39]. We report in particular an up−regulation of S100a8, S100a9, Prok2 (prokineticin 2/BV8) and Itgam (integrin alpha M/CD11b) in PyMT−Cxcr2−/− TANs compared to PyMT TANS, which could favor metastasis (Figure 6A). Pro−angiogenic factors such as MMP8 (matrix metalloproteinase−8), MMP9 (matrix metalloproteinase−9) and VEGFb (Vascular Endothelial Factor b) show also increased levels in PyMT−Cxcr2−/− TANs (Figure 6A), which is concomitant with an enrichment of VEGF receptor signaling as shown by GSEA analysis (Figure 6B). Several genes essential in the generation of reactive oxygen species (ROS), such as Arg2 (Arginase 2), Nos2 (Nitric oxide synthase 2) and S100a9 exhibited an up−regulation in PyMT−Cxcr2−/− TANs (Figure 6A), which is confirmed by the enrichment of reactive oxygen species metabolic process and response to oxidative stress by GSEA analysis (Figure 6C). In terms of cytokine production, there was an increase in G−CSF (Granulocyte Colony−Stimulating Factor/Csf3), a decrease of the chemokine CCL3 and TNFα levels (Figure 6A) and a depletion of Interferon signaling (Figure 6E). Interestingly, we also observed an inhibition of Myeloid Cell Apoptotic Process by GSEA analysis (Figure 6D). Altogether, this confirms the TAN2 features of PyMT−Cxcr2−/− TANs, which have potential impact on ROS production, metastasis, angiogenesis apoptosis, and neutrophil expansion.

### 3.9. PyMT−Cxcr2−/− TANs Exhibit a Defect in Tumor Cell Killing

**Figure 7:**
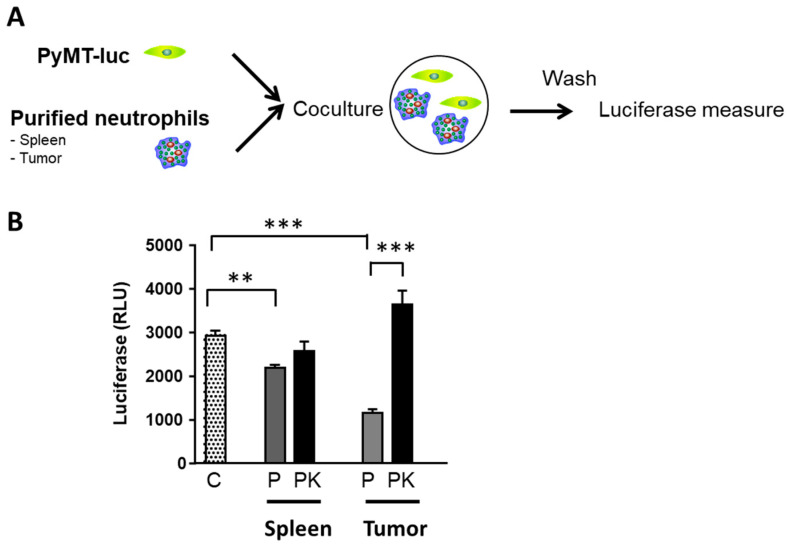
PyMT−Cxcr2−/− from tumors have no killing ability of PyMT cancer cells. (**A**) Neutrophils were isolated from the spleen or tumors of PyMT or PyMT−Cxcr2−/− animals. They were co−cultured with PyMT breast cancer cells expressing constitutively luciferase. After 24h, cells were washed and remaining PyMT cells were lysed for luciferase assay. (**B**) Quantification of luciferase activity of PyMT cells grown alone (C) or co−cultured with neutrophils isolated from the spleen or tumors of PyMT (P) or PyMT−Cxcr2−/− (PK) animals. Data represent the mean ± SEM of 18 wells from 2 independent experiments (Mann−Whitney test, ** *p*< 0.01, *** *p* < 0.001).

As we observed a more pronounced pro−tumor TAN2 profile of PyMT−Cxcr2−/− TANS compared to PyMT TANs, we wished to evaluate their tumor cell killing ability (Figure 7A). Briefly, PyMT cancer cells stably expressing a luciferase reporter gene were co−cultured with purified neutrophils from the tumor or spleen of PyMT and PyMT−Cxcr2−/− animals. The extent of killing was then measured after overnight incubation. We observed that spleen PyMT neutrophils had a weak ability to kill tumor cells (Figure 7B). On the contrary, spleen PyMT−Cxcr2−/− neutrophils did not affect tumor cells. More importantly, tumor PyMT neutrophils had a strong capacity to kill tumor cells, whereas tumor PyMT−Cxcr2−/− neutrophils did not (Figure 7B). These data confirm tumor PyMT−Cxcr2−/− TANs have TAN2 features, whereas PyMT TANs maintains anti−tumor ability, which could account for the higher rate of tumor growth in PyMT−Cxcr2−/− animals.

## 4. Discussion

The nature of immune infiltrate appears critical for the response of the host to tumor development, as it will enable or avoid immune escape. In this study, we showed, in an immune competent murine model of breast cancer (PyMT), that there was an increase in the levels of several ligands of Cxcr2 chemokine receptor. This is in agreement with the situation in human, as we and others previously shown that this was also the case for breast tumors compared to normal breast and for aggressive forms of breast cancer such as triple negative breast cancers (TNBC) compared to luminal breast cancers [7,8,10,11,12,41,42,43]. This increase of CXCR2 ligands is also observed for other types of cancers [9,44,45]. Moreover, we have recently shown that CXCR2 levels were also increased in human TNBC compared to luminal breast tumors [13], as shown for other types of cancers [46,47,48,49]. We find that Cxcr2 ablation leads to an increase of both primary tumor growth and the development of lung metastasis. This is in agreement with the work of Liu et al. [50], who reported that Cxcr2 ablation enhances tumor growth in a murine model of lung cancer. Moreover, we have also shown that high expression of CXCR2 in triple negative breast cancers was also a predictor of a lower risk of relapse [13,14], which confirms the protective role of Cxcr2. On the other hand, other studies have reported either no effect or inhibition on primary tumor growth, when Cxcr2 KO animals or Cxcr2 antibodies were used. For instance, genetic ablation of Cxcr2 in a transgenic model pancreatic cancer did not affect primary tumor growth, but inhibited metastasis [51]. In other types of models, Cxcr2−/− mice have been injected with lung cancer cells [52], breast cancer cells [53,54], pancreatic cancer cells [44], or renal cancer cells [55], and the authors have observed that deletion of Cxcr2 was reducing tumor growth. One major difference with these results and our work is that most studies involved injections of tumor cells to athymic mice or syngeneic mice and not a direct crossing of Cxcr2−/− animals with mice developing a cancer, recapitulating the complete tumor progression that we can observe in the PyMT model. Further, in contrast to most studies, which have crossed Cxcr2−/− mice with transgenic models of cancer [56] or initiated tumor onset by treating the mice with carcinogenic compounds [57,58], our mice were in a FVB background.

When comparing PyMT to WT glands, there was an increase of the number of neutrophils and macrophages, which is in agreement with previous studies [44,59,60,61]. Interestingly, when comparing PyMT−Cxcr2−/− to PyMT tumors, we observed a further increase in neutrophil infiltration, but a reduced number of macrophages. It is interesting to point out that tumor associated macrophages had a similar transcriptome in PyMT and PyMT−Cxcr2−/− animals (see Figure S8), suggesting that the main difference between macrophages in tumors was only their number and not their nature. The fact that Cxcr2 depletion could affect neutrophil recruitment is relevant to their high expression of Cxcr2. Moreover, in our hands, we have seen that neutrophils present the most prominent Cxcr2 levels compared to other types of cells (Figure S2). Of particular note, there was a 1.5 _fold increase in neutrophil content in the BM of PyMT−Cxcr2−/− animals compared to WT BM. In regard to the 45−fold increase of neutrophils in PyMT−Cxcr2−/− tumors compared to WT mammary, this suggests a preferential accumulation of neutrophils on PyMT−Cxcr2−/− tumors, which might be due to a higher time of retention and not due to an increased production of neutrophils by the BM.

We observed that PyMT−Cxcr2−/− TANs had a more pronounced TAN2 signature compared to PyMT TANs. This was confirmed by the fact that PyMT TANS were able to kill tumor cells, but PyMT−Cxcr2−/− TANs had lost this ability, which favors a TAN2 profile for PyMT−Cxcr2−/− TANs and could explain that these neutrophils are inefficient to counteract tumor growth.

Neutrophil polarization exists probably as a spectrum of activation sates, rather than as clearly defined states. The immunosuppressive functions of neutrophils are still not well understood. N1 anti−tumor cells are generally defined as mature cells, with cytotoxic, pro−apoptotic, anti−angiogenic, and stimulatory for T cells and immune activated cells, whereas N2 pro−tumor cells would be immature, anti−apoptotic, immune suppressive, and without stimulation [62]. Pro−tumor neutrophils are characterized by a high expression of Arginase, a low expression of TNFα, CCL3 and ICAM−1 according to Fridlender, which is effectively what we found [63]. To investigate in more detail the features of our TANs, we looked at a set of genes which have been reported by several studies as key players in the orientation of TAN1 versus TAN2 profiles. We identified several pathways that were altered in PyMT−Cxcr2−/− TANs. This includes first metastasis and neutrophil expansion. S100a8, S100a9, Prok2 were up−regulated and could favor metastasis [64], which is in agreement with the increased lung metastasis that we observed in PyMT−Cxcr2−/− animals. In the same line, CD11b, which was up−regulated, is involved in the guiding of cancer cells to metastatic sites [65]. Neutrophils achieve this by using neutrophil extracellular traps (NETs) to sequester circulating cancer cells [66]. Proteolytic enzymes MMP−8 and MMP−9, both increased, inactivate the tissue inhibitor of metalloprotease 1 (TIMP−1), and favor the invasiveness of cancer cells [67]. MMP9 is a key protease to remodel the extracellular matrix [68] and also has anti−apoptotic properties [69], which could account for the inhibition of apoptotic pathways that we observed by RNAseq analysis.

Moreover, MMP9 and Prok2 are pro−angiogenic factors, which also promote a leaky vasculature [70,71,72]. MMP9 produced by neutrophils promotes the angiogenic switch by inducing VEGF expression in the tumor [73] and we observed an up−regulation of VEGFR signaling.

S100a8 and S100a9 enhance the immunosuppressive activity of neutrophils and recruit immature myeloid cells to the tumor [74,75]. STAT3, which is also up−regulated, is one of the factors up−regulating S100a9 expression [75], and will contribute also to a higher production of ROS, which will increase the immunosuppressive action of neutrophils [76]. This is agreement with our GSEA analysis and the concomitant up−regulation of Arg2 and Nos2 in PyMT−Cxcr2−/− TANs. ROS will also favor genetic instability [27], which in turn will promote tumor progression.

We also observed several alterations in cytokine production in PyMT−Cxcr2−/− TANs. G−CSF, which expression is increased in PyMT−Cxcr2−/− TANs, is able to induce neutrophil generation and differentiation [77,78]. G−CSF promotes metastasis by controlling the immunosuppressive functions of the neutrophils [59]. G−CSF polarizes neutrophils towards a pro−tumor phenotype [59,64] and mobilizes neutrophils to pre−metastatic niches [79]. G−CSF also induces the release of Prok2 by neutrophils, which in turn promotes angiogenesis and cancer cell proliferation [80]. Prok2 stimulates also neutrophil expansion [70]. It is interesting to notice that neutrophils themselves are a source of G−CSF [81].

Interferon signaling was also down−regulated in PyMT−Cxcr2−/− TANs, which makes sense with the up−regulation of G−CSF that we observed, as type I IFNs downregulate G−CSF expression [81]. It has been reported that IFNβ deletion favors neutrophil infiltration, inhibits angiogenesis and favors tumor growth [72,82]. Moreover, Type I IFNs polarize neutrophils toward a N1 anti−tumor phenotype [62]. In the context of cancer, type I IFN play an anti−tumor role, by inhibiting proliferation and promoting apoptosis [83]. IFNβ is inhibiting VEGF and MMP9 production by TANs [72]. IFNβ also regulates the recruitment of neutrophils to the tumor and their longevity [81,84]. In addition, type II IFNγ produced by neutrophils enhances their ability to suppress T cell proliferation [85]. TANs with anti−tumor properties in the early stages of human lung cancer release IFNγ, which stimulates proliferation of T cells [86].

**Figure 8:**
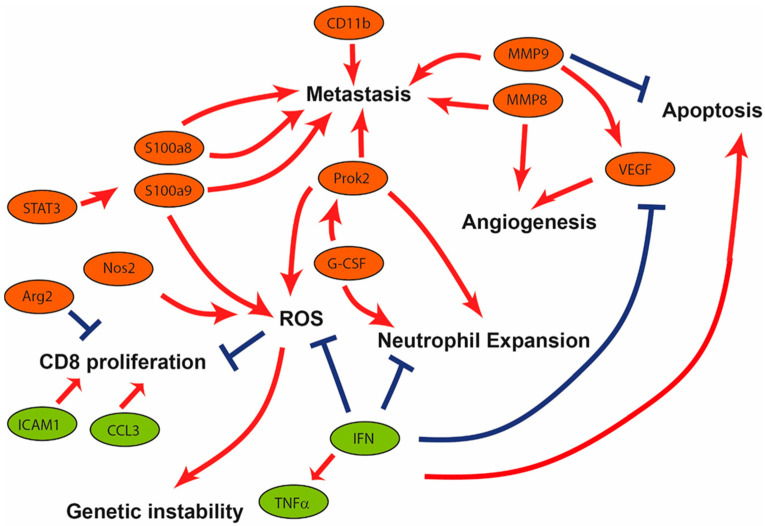
Scheme of the pathways affected in PyMT−Cxcr2−/− versus PyMT TANs. Representation of the differences observed in PyMT−Cxcr2−/− versus PyMT TANs. Genes in red boxes are up−regulated, whereas those in green boxes are down−regulated in PyMT−Cxcr2−/− versus PyMT TANs. Red arrows indicate stimulatory effects, whereas blue crosses indicate inhibitory signals. Factors such as CD11b, MMP8, MMP9, S100a8, S100a9 and Prok2 can increase metastasis. Several proteins including MMP8, MMP9 and VEGF can increase angiogenesis. Prok2, Nos2 and S100a9 can favor ROS generation, leading to a higher genetic instability. Moreover, Arg2 can inhibit CD8 proliferation, whereas the down−regulated genes coding for ICAM1 and CCL3, will be less prone to stimulate CD8 proliferation. G−CSF, which stimulates Pro2 production, will favor neutrophil expansion. Down regulation of IFN signaling, will enable an increase in neutrophil expansion, a higher production of ROS and a decreased apoptosis.

Altogether, the transcriptomic analysis of PyMT−Cxcr2−/− TANs reveals a TAN2 profile supported by the alteration of the production of several cytokines, which will modulate the levels of a number of factors involved in neutrophil expansion, metastasis, angiogenesis, ROS production, and apoptosis (Figure 8).

## 5. Conclusions

We could demonstrate that Cxcr2 is involved in the control of breast cancer development through the modulation of neutrophil composition within the primary tumor. Moreover, Cxcr2 ablation altered neutrophil properties, with Cxcr2−/− TANs showing a reduced anti−tumor ability associated with a more pronounced TAN2 transcriptome. This further reinforces the importance of Cxcr2 in neutrophil function in cancer progression and opens the door for a deeper analysis of Cxcr2 properties.

## Data Availability

The data discussed in this publication have been deposited in NCBI’s Gene Expression Omnibus [34] and are accessible through GEO Series accession number GSE164766 (https://www.ncbi.nlm.nih.gov/geo/query/acc.cgi?acc=GSE164766, accessed on 21 May 2021).

## Supplementary Materials

The following are available online at https://www.mdpi.com/article/10.3390/cancers13112584/s1, Figure S1: Cxcl1, 2, 3 and Cxcl5 levels increase in MMTV−Neu tumors, Figure S2: CXCR2 is expressed mainly by neutrophils, Figure S3: PyMT−Cxcr2−/− tumors grow faster than PyMT tumors at early and late stages, Figure S4: Measure of Cxcr2 ligands in the mammary gland and tumors, Figure S5: Representative graphs of, CD45+, CD11b+ and CD11b+ CD11c−Ly6Ghi Ly6Clo in tumors of PyMT and PyMT−Cxcr2−/− animals, Figure S6: Percentage of immune cells in the spleen, blood and bone marrow of PyMT and PyMT−Cxcr2−/− animals, Figure S7: Molecular analysis of neutrophil transcriptome, Figure S8: Molecular analysis of tumor associated macrophage (TAM) transcriptome, Table S1: Primers used for real−time PCR, Table S2: Percentage of immune cells among all cells of the mammary gland or tumor, Table S3: Comparison of the differences between PyMT and PyMT−Cxcr2−/− TANs with Shaul’s Signature, Table S4: Comparison of the differences between PyMT and PyMT−Cxcr2−/− TANs with Zilionis’s Signature.
