# Supplementary material for: Pivotal Role for Cxcr2 in Regulating Tumor-Associated Neutrophil in Breast Cancer
Source: Cancers (Basel). 2021 May 25;13(11):2584. doi: 10.3390/cancers13112584 (PMC8197482; doi:10.3390/cancers13112584)
Supplement: Supplementary file 1 [file cancers-13-02584-s001.zip › cancers-1206161-supplementary.pdf]

cancers MDPI

Supplementary Materials

Pivotal Role for Cxcr2 in Regulating Tumor−Associated Neutrophil in Breast Cancer

Colin Timaxian, Christoph F. A. Vogel, Charlotte Orcel, Diana Vetter, Camille Durochat, Clarisse Chinal, Phuong NGuyen, Marie−Laure Aknin, Françoise Mercier−Nomé, Martin Davy, Isabelle Raymond−Letron, Thi−Nhu−Ngoc Van, Sarah D. Diermeier, Anastasia Godefroy, Magali Gary−Bobo, Franck Molina, Karl Balabanian and Gwendal Lazennec

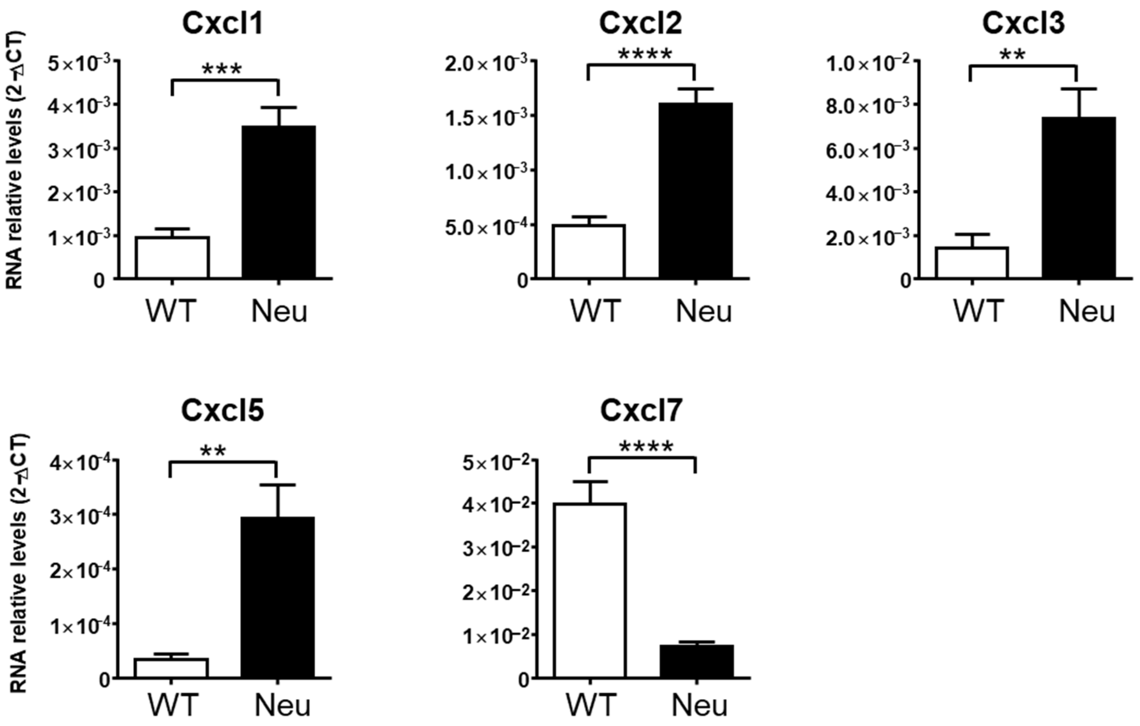

Figure S1. Cxcl1, 2, 3 and Cxcl5 levels increase in MMTV−Neu tumors. Measure of RNA levels by real−time PCR of Cxcl1, 2, 3, 5 and 7 in the mammary gland of WT animals or the tumors of MMTV−Neu {Guy, 1992 #1098} animals. Results represent the mean ± SEM of at least 10 animals (Mann−Whitney test, ** $p < 0.01$, *** $p < 0.001$, **** $p < 0.0001$).

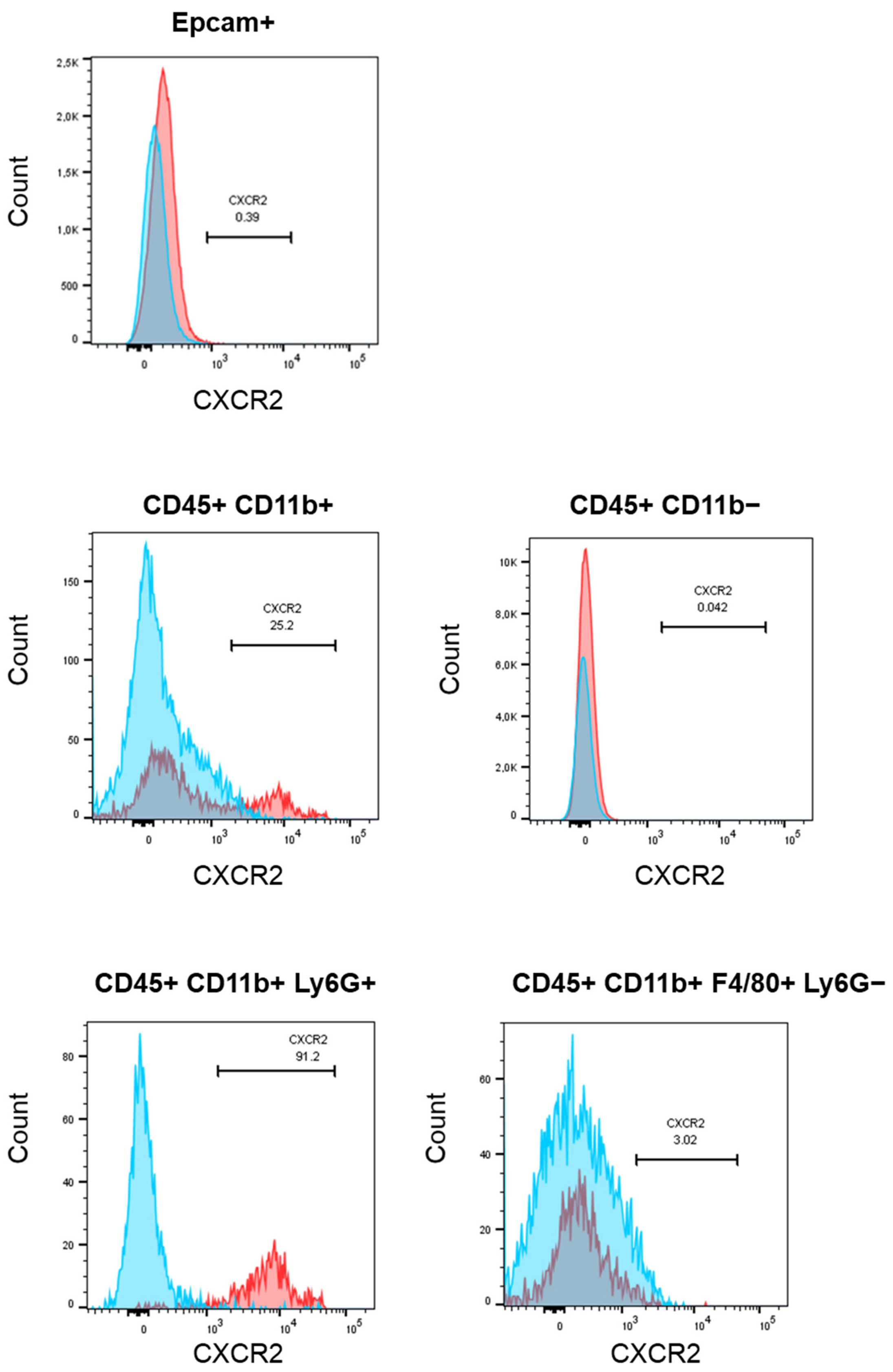

Figure S2. CXCR2 is expressed mainly by neutrophils. Representative flow charts of CXCR2 labelling of PyMT (red histograms) or PyMT− Cxcr2−/− (blue histograms) tumors. Upper panel: measure of CXCR2 in epithelial cancer cells (Epcam+ cells). Middle panel: measure of CXCR2 in CD45+ CD11b+ granulocytic cells (left panel) or CD45+ CD11b− (right panel). Bottom panel: measure of CXCR2 in neutrophils (CD45+ CD11b+ Ly6G+ cells) (left panel) or macrophages (CD45+ CD11b+ Ly6G− F4/80+ Ly6G− cells) (right panel).

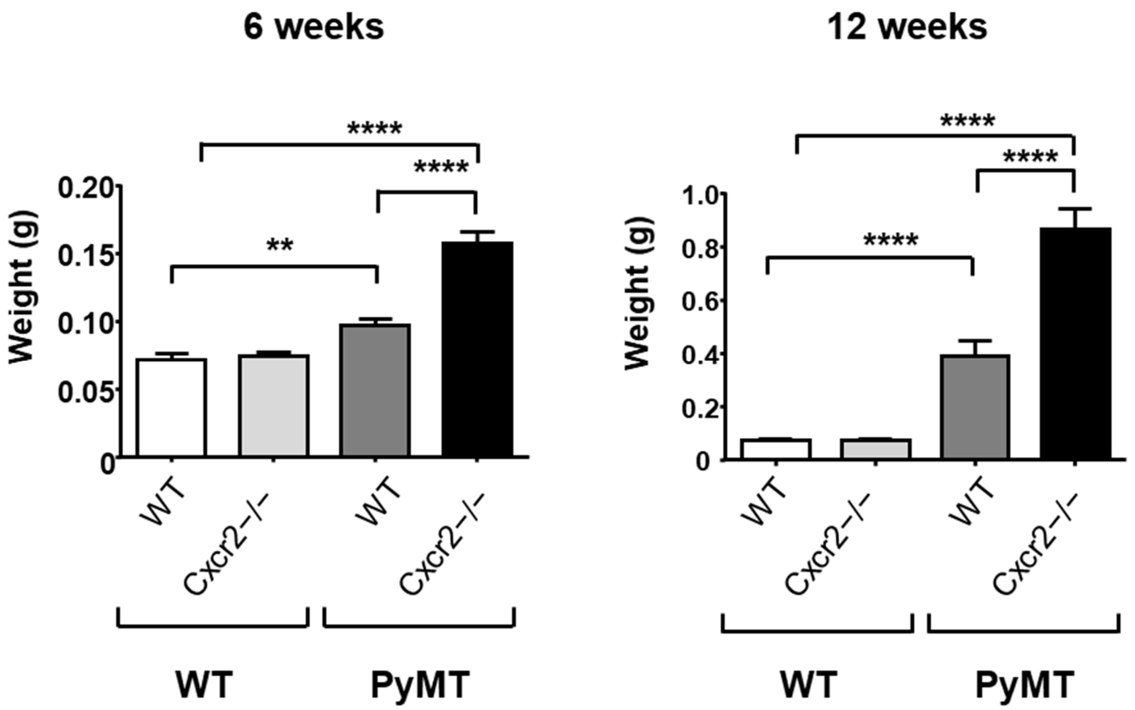

Figure S3. PyMT−Cxcr2−/− tumors grow faster than PyMT tumors at early and late stages. Weight of the mammary gland of WT, Cxcr2 KO, PyMT and PyMT−Cxcr2−/− mice at 6 weeks (left panel) and 12 weeks (right panel). Results represent the mean ± SEM of at least 18 animals (Mann−Whitney test, ** $p < 0.01$, **** $p < 0.0001$).

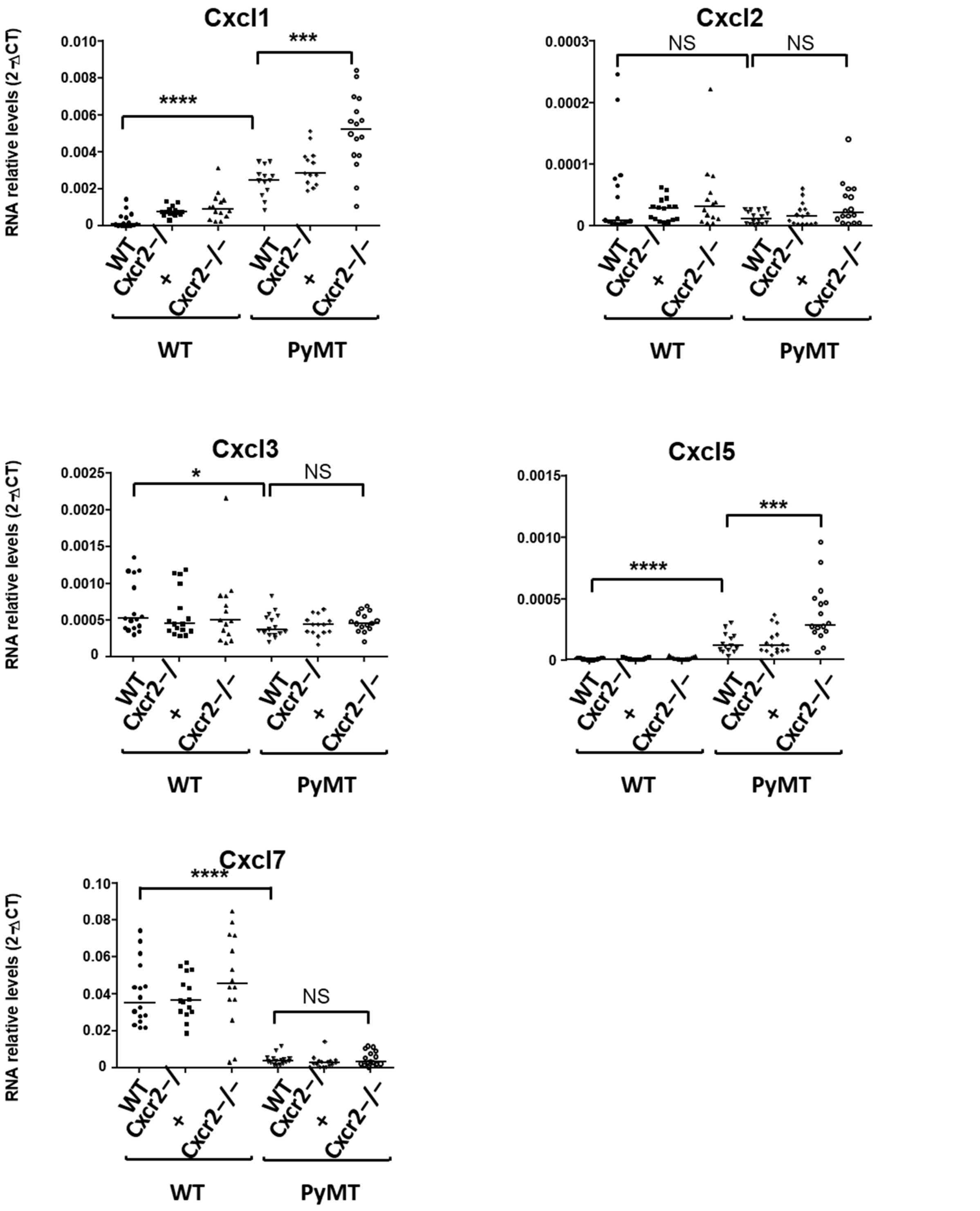

Figure S4. Measure of Cxcr2 ligands in the mammary gland and tumors. Measure of RNA levels by real−time PCR of Cxcl1, 2, 3, 5 and 7 in the mammary gland or mammary tumor of WT, Cxcr2−/+, Cxcr2−/−, PyMT, PyMT−Cxcr2−/+ and PyMT−Cxcr2−/− animals aged of 10 weeks. Results represent the mean ± SEM of at least 14 animals (Mann−Whitney test, NS: non−significant, * $p < 0.05$, *** $p < 0.001$, **** $p < 0.0001$).

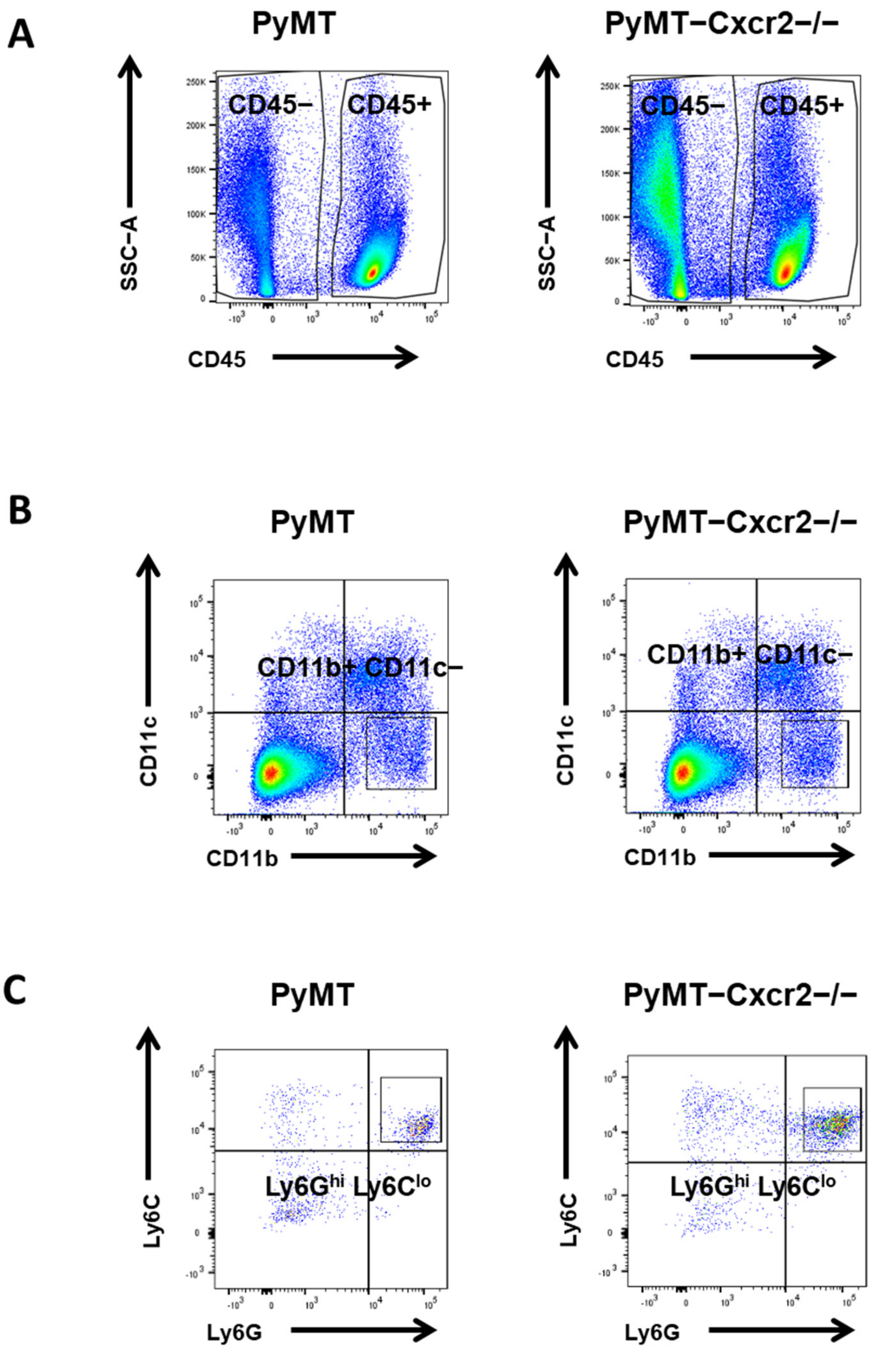

Figure S5. Representative graphs of, CD45+, CD11b+ and CD11b+ CD11c−Ly6G^{hi} Ly6C^{lo} in tumors of PyMT and PyMT−Cxcr2−/− animals. (**A**) Representative flow cytometry graphs of CD45− and CD45+ cells in PyMT and PyMT−Cxcr2−/− tumors. (**B**) Representative flow cytometry graphs of CD45+ CD11b+ CD11c− cells (right lower quadrant) in PyMT and PyMT−Cxcr2−/− tumors. (**C**) Representative flow cytometry graphs of CD11b+ CD11c−Ly6G^{hi} Ly6C^{lo} cells (right upper quadrant) in PyMT and PyMT−Cxcr2−/− tumors.

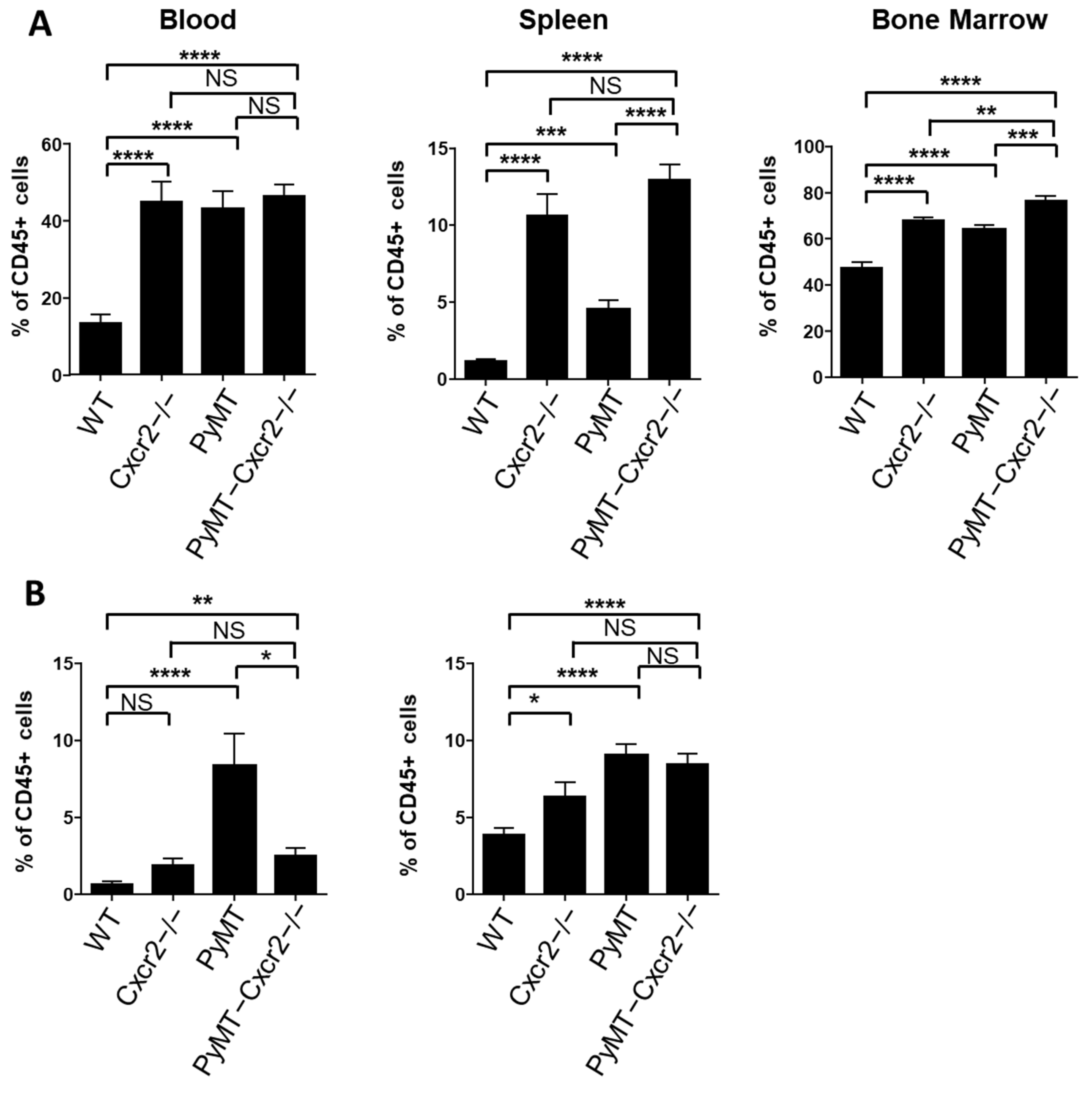

Figure S6. Percentage of immune cells in the spleen, blood and bone marrow of PyMT and PyMT−Cxcr2−/− animals. (**A**) Quantification of CD11b+ CD11c−Ly6G^{hi} Ly6C^{lo} neutrophils in the CD45+ fraction of blood (left panel), spleen (middle panel) and bone marrow (right panel). Data represent the mean ± SEM of at least 7 animals (Mann−Whitney test, NS: non−significant, * $p < 0.05$, ** $p < 0.01$, *** $p < 0.001$, **** $p < 0.0001$). (**B**) Same analysis of CD11b+ Ly6G− F4/80+ macrophages in the CD45+ fraction in blood (left panel) and spleen (right panel).

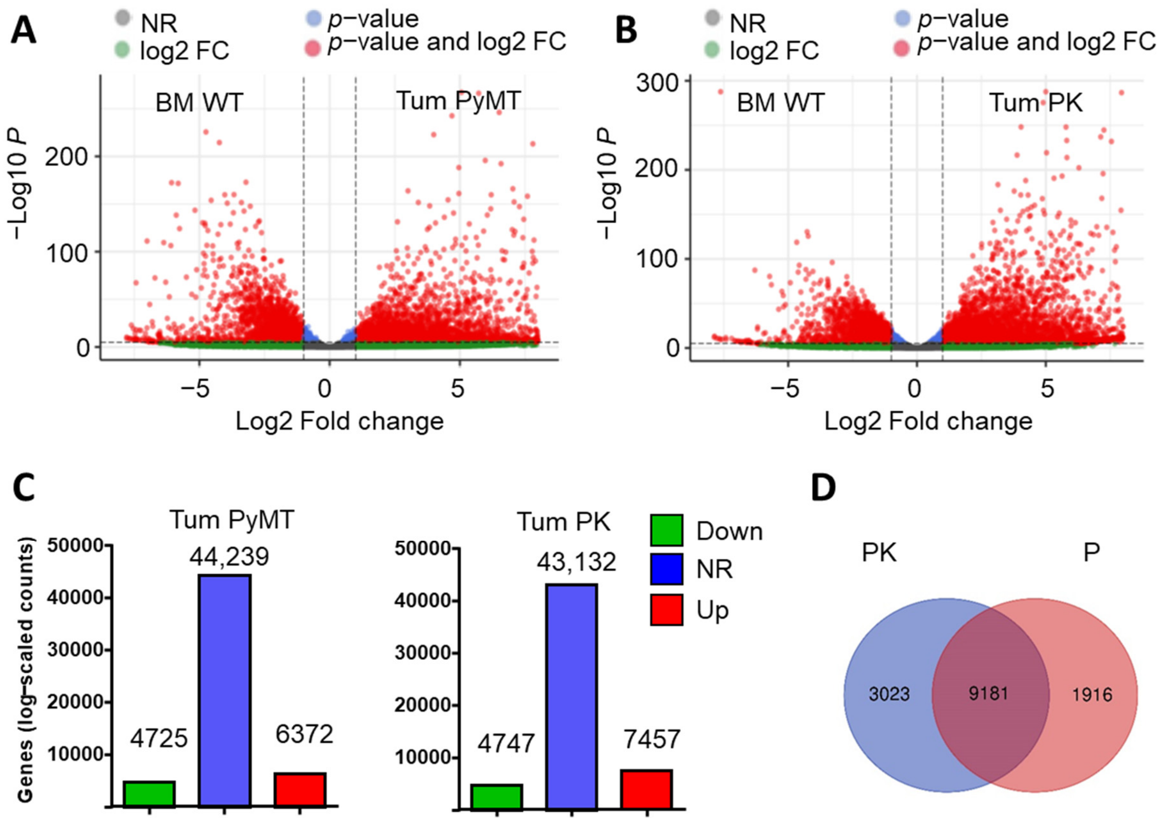

Figure S7. Molecular analysis of neutrophil transcriptome. (**A**) Volcano plot showing the global changes in RNA expression patterns for Tumor neutrophils isolated from PyMT animals compared to BM neutrophils from WT animals. Data represent analysis of cpm estimates with a log of fold change of more than 1.5 fold and $p < 0.05$ of 4 animals per group. Grey dots: NR: non−regulated genes; Green dots: genes with a log of fold change of more than 1.5 fold; blue dots: genes with a p−value < 0.05; red dots: genes with a log of fold change of more than 1.5 fold and $p < 0.05$. (**B**) Same analysis of Tumor neutrophils isolated from PyMT−Cxcr2−/− versus BM neutrophils from WT animals. (**C**) Left Panel: Number of differentially regulated genes for the analysis presented in A. Up: genes up−regulated in Tumor neutrophils isolated from PyMT animals versus neutrophils from BM WT animals. Down: down−regulated genes. NR: non−regulated genes. Right Panel: Number of differentially regulated genes for the analysis presented in B of Tumor neutrophils isolated from PyMT−Cxcr2−/− versus BM neutrophils from WT animals. (**D**) Venn diagram of common differentially regulated genes between PyMT−Cxcr2−/− (PK) versus BM and PyMT (P) versus BM neutrophils.

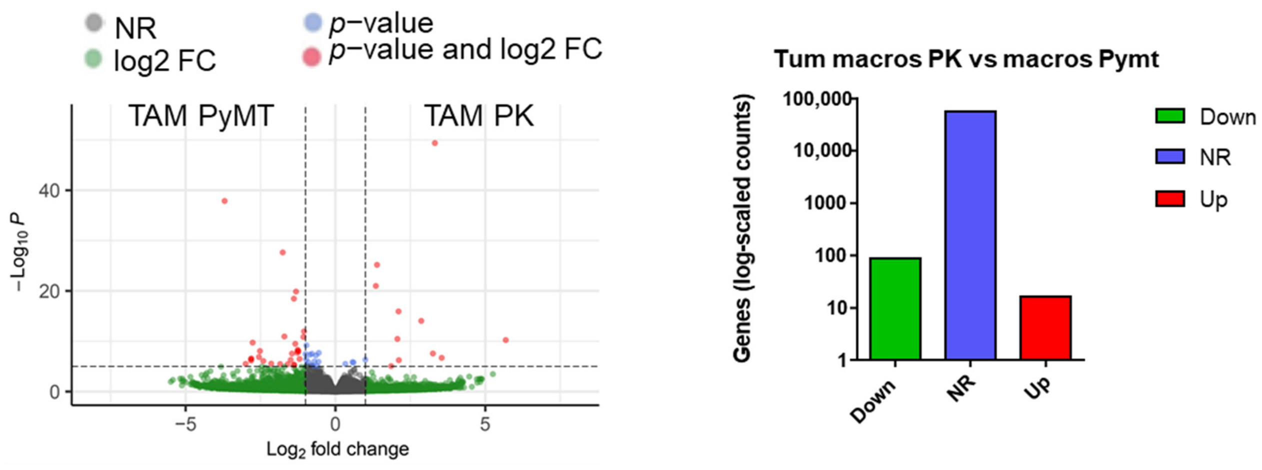

Figure S8. Molecular analysis of tumor associated macrophage (TAM) transcriptome. Left panel: Volcano plot showing the global changes in RNA expression patterns for Tumor macrophages isolated from PyMT animals compared to those of PyMT−Cxcr2−/− animals. Data represent analysis of cpm estimates with a log of fold change of more than 1.5 fold and $p < 0.05$ of 4 animals per group. Grey dots: NR: non−regulated genes; Green dots: genes with a log of fold change of more than 1.5 fold; blue dots: genes with a p−value < 0.05; red dots: genes with a log of fold change of more than 1.5 fold and $p < 0.05$. Right panel. Number of differentially regulated genes for the analysis presented in left panel. Up: genes up−regulated in Tumor macrophages isolated from PyMT−Cxcr2−/− versus PyMT animals. Down: down−regulated genes. NR: non regulated genes.

Table S1. Primers used for real−time PCR.

Gene	Upper Primer Sequence	Lower Primer Sequence
Gapdh	GGTGCTGAGTATGTCGTGGA	GTGGTTCACACCCATCACAA
Rs9	CGGCCCGGGAGCTGTTGACG	CTGCTTGCGGACCCTAATGTCACG
CXCL1	AACGCTGGCTTCTGACAACACTAT	CTTTTCGCACAACACCCTTCTACT
CXCL2	TTGCCCAGATGTTGTTATGTTTAT	CCACCCCCACCCCTTATCC
CXCL3	ACCGCGCCCTGCCTAGAAAAGAC	CAAAAGCCTCCCAATCCAAACAT
CXCL5	CGGAGCTGCGTTGTGTTTG	CATTCCGCTTAGCTTTCTTTTTGT
CXCL7	TGCGCTGGCTCCCCTTACA	TGGCTTGCCCGTCTTCATCAT

Table S2. Percentage of immune cells among all cells of the mammary gland or tumor.

	WT	Cxcr2−/−	PyMT	PyMT-Cxcr2−/−
% of CD11b+ CD11c− cells	0.69 ±0.11	1.85 ± 0.55	1.27 ± 0.33	3.15 ± 0.94
% of CD11b+ CD11c Ly6G^{hi} Ly6C^{lo} cells	0.32 ± 0.07	0.90 ± 0.26	0.57 ± 0.17	2.42 ± 0.82
% of CD11b+ Ly6G− F4/80+ cells	0.57 ± 0.21	1.01 ± 0.38	9.24 ± 3.4	3.80 ± 0.85

Table S3. Comparison of the differences between PyMT and PyMT-Cxcr2−/− TANs with Shaul's Signature.

Pathway	padj	NES	Size	Genes
TAN2 vs TAN1	0.0037	1.935	387	*MPZL3,CCNO,ST3GAL5,RALGPS1,E2F2,KIAA0513,RNF144A,DOCK8,CYFIP2,MYO1G,CD3D, DGKG,NCOA1,ABTB1,SAMHD1,TMEM154,SATB1,DACH1,THBD,PI16,IPCEF1,USP2,TBC1D8, ST8SIA4,HAAO,FAM160A2,RAB27A,FBXO31,MOCOS,ABCA7,GDI1,DENND3,TXNL4B,SH2D3C, NDST1,SLC44A2,GSDME,INLR1,LINS1,SIPA1L1,GMIP,MOB3A,CCDC88B,CDK5R1,AKNA, ARID3B,UBE3B,SNRK,PLEKHM3,OGT,P2RY1,SETX,IL18R1,NOCT,STAT3,MGST2,ARL5B,ACTA2, MSRB1,TRIP12,VPS26B,DAB2IP,LPGAT1,MRPS28,BIRC3,NLRX1,CKLF,KHNYN,TMEM164,CMC2, SASH3,IRAK4,ARHGAP25,IKZF1,ZC3H12A,PRKD3,RGS14,SLC25A37,CDC42EP3,SSH2,TNRC6C, PPP1R15B,SLC16A6,VAMP4,TRPS1,JOSD2,AGRN,CCR7,INTS3,MBP,BIN3,KLHDC4,CDK19,SPATC1, SLCO3A1,IST1,F2RL2,CD6,VPS4B,PIP5K1A,SYN1,ITPR2,NUP210,CYP2S1,NOXRED1,FAM102A,RIN3, NT5E,BCL6,PHLDA1,DPP4,AQP9,GDF3,C1GALT1,MED12,CD300A,CD300LD,IL16,RNASE2,RNASE3, AREG,ACOT1,ACOT2,PTPRC,BOLA2,BOLA2B,GPR183,NARF,FBXL18,PRR15,ACAM,ARHGAP22, CTC1,MAPKAPK3,DNAJC4,PDLIM7,PTPRS,C5orf34,SCAP,CDC42EP4,KCNN4,RDH5, DCTAMP,PLIN5,CIITA,AMMECR1L*

Table S4. Comparison of the differences between PyMT and PyMT−Cxcr2−/− TANs with Zilionis's Signature.

Pathway	padj	NES	Size	Genes
Nm1	0.037	3.361	182	*S100A6,MMP8,PADI4,NHSL2,STX11,ZNF516,SLC16A3,SCRG1,PGLYRP1,SLC2A6,ANXA1, S100A11,SVIL,XDH,R3HDM4,HACD4,ST3GAL5,E2F2,ARL2BP,PHACTR2,CPNE2,PALM, KIAA0513,RNF144A,AC010616.1,ATP1A3,MTUS1,S1PR4,GDA,STK10,MMP9,KDM7A,LIMD2, CAPN1,MAP3K15,DGAT1,RCSD1,FPR1,MGST1,SLC66A3,CHST12,PLBD1,XPO6,NCOR1, ALOX5AP,PBX2,RERE,MSL1,CCND3,PCMTD2,NBEAL2,DCK,FAM160A2,ARAP3,LILRA4, LILRA5,LILRA6,LILRB3,LILRB5,OBPL2,PTEN,KCTD12,GDI1,RDH12,ARHGAP30,DLGAP4, CELF2,MFSD6,INPP1,GPI,CLIP1,NEK9,EMB,ARAP1,CDK5R1,SLC44A1,ZYX,GSR,MYADM, CD37,SLPI,SNRK,ANKRD44,METTL9,GFOD1,CEACAM1,CEACAM3,CEACAM5,CEACAM6, CEACAM7,CEACAM8,ZXDC,RENLB,ULK1,SLC22A15,HDAC4,CHSY1,HMGB2,LPCAT4, STAT5B,TNFRSF21,ACTA2,WBP1L,MAP3K5,TCP11L2,MRPL33,FAR1,PHOSPHO1, KIAA0930,INPP5K,H33A,LPCAT2,PHF1,FAM32A,C19orf38,TMPO,APBB1IP,SCNN1A, RNF125,COQ10B,IP6K1*
Nm6	0.037	2.152	155	*LTF,RAB3D,LCN2,CD177,TKT,SH3KBP1,MEGF9,ACTN1,DOCK8,CAMP,S100A8,MYO1G, ANXA3,PLCG2,ABCA13,DACH1,DOK2,PAK1,KCNAB2,PYGL,CYBB,INSR,BST1,C1RL, SMPDL3A,TPST2,SLC39A11,SHC1,ALOX5,B4GALT6,CD101,CTNNBIP1,TLE4,MGRN1,APRT, ZFHX3,SERPINB1,GTPBP1,AOAH,RPS6KA1,ETHE1,CHIA,SEMA7A,ENTPD6,ANTXR2, FCN1,GSN,ITPRIPL2*
Nm3	0.0274	−1.474	125	*CXCR2,HIC1,JAK2,EGLN3,RHOH,HAVCR2,P4HB,BCOR,TGM2,CPEB2,CISH,CLEC5A, IER3,EML4,MED7,XBP1*
Nm5	0.0118	−1.485	193	*COL15A1,CCL3L3,CCL3,CCL18,ITGAE,CSTB,CXCR1,MREG,ST13,HILPDA,GNL3,ILF3, NAA50,IGF2R,AARS1,RALGDS,AL162417.1,CIART,FRRS1,CITED2,MCFD2,GAS2L3,VWF,CD63, C15orf39,CLCN7,HSP90AA1,CNOT4,SNX8,RAB9A,BAX,F10,TRPM7,SIRT1,MGAT4B,ARL8B, FNIP2,DNAJB9,EEA1,SFT2D3,SH2B2,CCL4L2,CCL4,FAM167A,DGKZ,STX3,F7,SDCBP,FNIP1*
Nm4	0.0087	−1.813	180	*NSF,CDKN1A,DHX15,PTGS1,U2AF1L5,U2AF1,DAD1,ENTPD3,TARDBP,ZFP28,CNBP,ISYNA1, BANF1,BOP1,AAAS,EXOC4,PRPS1,GPR171,SSBP1,CYSLTR1,SSRP1,SNRPD3,AP000356.5, GNGT2,TOMM7,ALYREF,NDUFB4,HNRNPA2B1,FZD7,NAAA,MRPL42,TOR1B,HMGN5, KRTCAP2,RWDD1,EI24,GLRA1,NUP153,C12orf57,KDM1B,PRPF8,NDUFA2,IL1RL2,RBM12B, DYNLL1,ATP5PF,ELP3,NDUFB5,SRP72,NXT1,G6PC3,RNF220,NDUFAF3*
